# Zinc metabolism and its role in immunity status in subjects with trisomy 21: chromosomal dosage effect

**DOI:** 10.3389/fimmu.2024.1362501

**Published:** 2024-04-17

**Authors:** Giuseppe Ramacieri, Chiara Locatelli, Michela Semprini, Maria Chiara Pelleri, Maria Caracausi, Allison Piovesan, Michela Cicilloni, Marco Vigna, Lorenza Vitale, Giacomo Sperti, Luigi Tommaso Corvaglia, Gian Luca Pirazzoli, Pierluigi Strippoli, Francesca Catapano, Beatrice Vione, Francesca Antonaros

**Affiliations:** ^1^ Department of Medical and Surgical Sciences (DIMEC), University of Bologna, Bologna, Italy; ^2^ Specialist School of Child Neuropsychiatry - University of Bologna, Bologna, Italy; ^3^ Neonatology Unit, St. Orsola-Malpighi Polyclinic, Bologna, Italy; ^4^ Unit of Histology, Embryology and Applied Biology, Department of Biomedical and Neuromotor Sciences (DIBINEM), University of Bologna, Bologna, Italy; ^5^ Speciality School of Paediatrics - Alma Mater Studiorum, University of Bologna, Bologna, Italy; ^6^ Neonatology Unit, Department of Medical and Surgical Sciences (DIMEC), St. Orsola-Malpighi Polyclinic, University of Bologna, Bologna, Italy; ^7^ Medical Department, Maggiore Hospital, Bologna, Italy

**Keywords:** down syndrome, zinc, immunity disorder, transcriptome, integration

## Abstract

**Introduction:**

Trisomy 21 (T21), which causes Down syndrome (DS), is the most common chromosomal aneuploidy in humankind and includes different clinical comorbidities, among which the alteration of the immune system has a heavy impact on patient’s lives. A molecule with an important role in immune response is zinc and it is known that its concentration is significantly lower in children with T21. Different hypotheses were made about this metabolic alteration and one of the reasons might be the overexpression of superoxide dismutase 1 (*SOD1*) gene, as zinc is part of the SOD1 active enzymatic center.

**Methods:**

The aim of our work is to explore if there is a linear correlation between zinc level and immune cell levels measured in a total of 217 blood samples from subjects with T21. Furthermore, transcriptome map analyses were performed using Transcriptome Mapper (TRAM) software to investigate whether a difference in gene expression is detectable between subjects with T21 and euploid control group in tissues and cells involved in the immune response such as lymphoblastoid cells, thymus and white blood cells.

**Results:**

Our results have confirmed the literature data stating that the blood zinc level in subjects with T21 is lower compared to the general population; in addition, we report that the T21/control zinc concentration ratio is 2:3, consistent with a chromosomal dosage effect due to the presence of three copies of chromosome 21. The transcriptome map analyses showed an alteration of some gene’s expression which might explain low levels of zinc in the blood.

**Discussion:**

Our data suggest that zinc level is not associated with the levels of immunity cells or proteins analyzed themselves and rather the main role of this ion might be played in altering immune cell function.

## Introduction

Trisomy 21 (T21), which causes Down syndrome (DS) (OMIM 190685) (1), is the most common chromosomal aneuploidy in humankind and it is usually associated with a delay in cognitive ability and physical growth, as well as a particular set of clinical features (2).

One of the clinical comorbidities of subjects with DS is the alteration of the immune system. The adaptive and innate immune responses in individuals with trisomy 21 are aberrant at multiple levels, including dysfunction of the cellular and humoral responses, altered phagocytic function of myeloid cells, partial deficiency of complement proteins, increased cytokine responses (3, 4) elevated interferon (IFN) signaling (5, 6), and altered toll-like receptor (TLR) signaling (7). In fact, children with trisomy 21 are a high-risk group who get more severe infections and have poorer outcomes, with a higher overall mortality rate; they are more likely to be admitted to hospital, have an increased length of stay due to respiratory tract infection and a greater chance of requiring ventilatory support and intensive care (7–10).

Verstegen et al. hypothesize three molecular mechanisms that can affect cellular development function and survival: the direct effect of dysregulated gene expression, impaired cellular communication and local environment (i.e., pro-inflammatory cytokine profile, chronic interferon activity, increased oxidative stress conditions) (10).

One chemical element with an important role in the immune response is zinc (Zn). It is involved in the maintenance and development of both innate and adaptive immune systems (11), and its deficiency results in an altered host defense, increased risk of inflammation and even death. In subjects with DS a decreased concentration of this element in serum, plasma and whole blood is reported (12, 13).

Zinc is essential for life and is the second most abundant transition metal ion in living organisms after iron. It is found predominantly bound to albumin (60%, low-affinity), α2-macroglobulin (30%, high-affinity) and transferrin (10%) (14–16), which help to maintain a proper concentration (17). In contrast to iron, zinc cannot be stored and must be taken up via food daily to guarantee sufficient supply (17).

A disrupted zinc homeostasis leads to weakened innate host defense via phagocytosis and oxidative burst, impaired formation, activation, and maturation of lymphocytes, including thymic atrophy, and disturbed intercellular communication via cytokines (18). Moreover, through the interaction of zinc fingers with homeoboxes (ZHX) and transcription factors, zinc regulates gene expression (19).

According to meta-analysis by Barišić and Saghazadeh, zinc concentration was significantly lower in serum, plasma and whole blood of children and adolescents with trisomy 21 compared to control group (12, 13) instead copper (Cu) and zinc levels were higher in trisomic red blood cells (12). This meta-analysis showed that females and males with trisomy 21 do not differ in Zinc levels and evidence was not conclusive with regards to the effect of age on zinc levels (12).

One of the reasons for the zinc alteration in subjects with DS might be the overexpression of superoxide dismutase 1 (*SOD1*) gene (20), located on chromosome 21. *SOD1* has been found to be expressed 50% higher than normal in various cells and tissues of people with trisomy 21 (21). Cu-Zn-SOD is an intracellular dimeric enzyme containing Cu and Zn ions in the active site (22). Considering that zinc is part of the SOD1 active enzymatic center, it seems very likely that the elevated zinc and copper levels in red blood cells of subjects with trisomy 21 are due to the increased intraerythrocytic levels and activity of SOD1 (23). Nevertheless, the increase of SOD1 activity could also lead to excessive zinc consumption because it is part of the active enzymatic center (13).

Metallothioneins, a family of 19 cysteine-rich, low molecular weight proteins, are located in the membrane of the Golgi apparatus and have a known antioxidant role. Any changes related to these proteins are reflected in altered zinc concentrations such as higher levels of metallothionein 3 in trisomic astrocytes have been shown to cause a lower concentration of free zinc (24).

Solute carrier family 30 (*SLC30*) and 39 (*SLC39)* produce protein transporters involved in zinc homeostasis, functioning in opposite directions. SLC30 proteins maintain cytoplasmic zinc balance exporting zinc out or sequestrating cytoplasmic zinc into intracellular compartments when cellular concentration is elevated. SLC39 proteins increase the cytoplasmic zinc concentration importing the ion in when cellular concentration is depleted (25).

The effectiveness of zinc supplementation in people with trisomy 21 has been reported with discordant results. It is reported from several authors that zinc sulphate supplementation has a positive effect in increasing zinc serum levels and thymulin plasma levels (26–28), normalizing neutrophil chemotaxis and lymphocyte response to phytohemagglutinin, increasing the absolute number of circulating T lymphocytes, and decreasing infections (26, 29). Other authors, who administered zinc gluconate in children with trisomy 21 obtained a tendency towards a decrease of days or episodes of cough and fever but without any effect on other clinical items, such as immunoglobulin and complement serum levels, the number of lymphocytes, or the *in vitro* response to mitogens (30). In another work, no correlation was found between low zinc levels in a cohort of children with trisomy 21 and the recurrence and/or the intensity of infections (29).

It has been shown that zinc supplementation enables DNA repair in lymphocytes of people with trisomy 21 (31). Indeed, a significant increase in the synthesis of DNA was obtained after the administration of zinc sulphate orally.

It is relevant to report that zinc sulphate treatment seems to induce a clonal selection of immature and ineffective peripheral myeloid cells by a zinc-induced cell death mechanism (32). Leukocytes contain high levels of zinc and levels vary with cellular maturity, lowest being in the most immature cells; low plasma zinc levels shown in subjects with trisomy 21 could be responsible for the inhibition of normal differentiation of myeloid cells and for undifferentiated cells coming out from bone marrow to peripheral blood (32).

The aim of our work is to confirm literature data on zinc levels in subjects with T21 in our T21 cohort compared to a control group in a larger cohort of subjects with trisomy 21, then to understand if there is a linear correlation between zinc levels and immune cell levels and finally to search if there is any difference in gene expression in lymphoblastoid cells, thymus and white blood cells from subjects with trisomy 21 and control group subjects.

## Materials and methods

### Ethics statement

The ethical approval for this study has been granted by the Independent Ethics Committee of the IRCCS University Hospital Company of Bologna, Policlinic of Sant’Orsola, Italy (number: 39/2013/U/Tess). Informed written consent has been obtained from all participants to collect blood samples and clinical data as well as for genetic analysis; if the subject was below 18 years of age, the consent was collected from parents or legal tutor.

### Down syndrome case selection

Blood samples and clinical data were collected in the context of the yearly routine follow up provided for patients with trisomy 21 at the Day Hospital of the Neonatology Unit in IRCCS University Hospital Company of Bologna, Policlinic of Sant’Orsola. Sample collection and data analysis for research started in 2014 within the “Genotype-phenotype correlation in trisomy 21 (Down syndrome)” project.

For the aim of this study, the inclusion criteria for children were diagnosis of trisomy 21 and no zinc supplement, antibiotic or oral cortisone therapy at blood draw (local cortisone therapy was allowed). A total of 217 subjects with trisomy 21 were recruited in this study; one patient was excluded because the zinc value (34.72 µmol/L) was an outlier in the group of enrolled subjects. This exclusion makes the total number of patients included in **Supplementary Dataset 1** file 216 (from age 10 months to 34 years). In particular we included 30 babies between 10 months and 4 years old, 77 kids between 4 and 9 years old, 59 children between 9 and 14 years old, 25 teenagers between 14 and 18 years old and 25 adults over 18 years old. Some blood values of immunity data are missing due to an inadequate volume of blood obtained or due to problems occurred during laboratory analysis, or not considered in the statistical analysis because identified as strong outlayer (highlighted in red in **Supplementary Dataset 1**). We excluded from the analysis 7 subjects without information on fasting state (Fasting state=N/A) when the metabolite levels were influenced by fasting state. Because of this we indicated for each statistical analysis the total amount of patients studied. Subjects with pathological lymphopenias or lymphocytosis were not present in the population and thus discussion of the results refers only to situations within physiological limits. For every collected sample, information has been gathered regarding current fasting state, most recent meal and drugs taken.

All data can be found in **Supplementary Dataset 1**.

### Preparation and analyses of T21 blood samples

Blood analyses were performed by Laboratorio Unico Metropolitano of Bologna (LUM) in the contest of the yearly routine follow up provided for patients with T21. Blood samples were collected in EDTA-coated blood collection tubes and kept at room temperature and were treated within 2 hours from blood draw.

The quantitative determination of zinc (µmol/L) in human serum was detected by direct colorimetric method without the deproteinization step on Beckman AU analyzer. The quantitative determination of percentage (%) of total lymphocytes was detected in human serum using a turbidimeters measurement on Beckman Coulter AU analyzer: immunoglobulin A (IgA) (OSR61171), IgG (OSR61172), and IgM (OSR61173). Complete blood count (CBC) (103/mmc), including white blood cells, basophils, neutrophils, lymphocytes, monocytes, and eosinophils was performed by fluorescent flux cytometry using Sysmex XN technology. The lymphocyte typing (T cells, T CD4+ cells, T CD8+ cells, B cells, natural killer cells) was performed on whole blood using BD FACSCanto II flow cytometry (BD Biosciences) and analyzed with BD FACSDiva software (BD Biosciences).

### Control data selection

A bibliographic search was done on the National Center for Biotechnology Information (NCBI) PubMed website in order to find a control (CTRL) population that matched as closely as possible in age, historical period, and geographic location.

López’s 1997 article seemed to be the most suitable, having analyzed zinc levels in a healthy Spanish pediatric population of 3668 subjects aged from 4 to 18 years old (33). The subjects were selected randomly from child and adolescent populations in Navarra, Spain in the context of an epidemiological study on cardiovascular risk factors. Zinc was analyzed in the serum collected in 1987 and stored in polypropylene containers at -20°C for five years (33). López in his study described a normal distribution and reported number of cases, mean and 95% confidence interval for male and female of each age from 4 to 18 years old (33). We summarized his data in **Supplementary Table 1**. In addition, López’s 1997 work, which was used as control, also excluded values above or below 2 standard deviation (SD), considering them as outliers (33). We obtained the SD from 95% confidence interval from the work of López 1997 using the method that can be found in **Supplementary File 1**.

### Statistical analyses

All statistical analyses were performed using SPSS Statistics (IBM software) except for the student’s t-test analysis between subjects with T21 and CTRL for which we used GraphPad online software (https://www.graphpad.com/quickcalcs/ttest1.cfm). The Kolmogorov-Smirnov test was executed using an online tool (https://www.socscistatistics.com/tests/kolmogorov/default.aspx) to know if the data follow a normal distribution. Strong outliers were excluded from the analyses.

For all results, p<0.05 was required for statistical significance. When R or Cramer’s V coefficient was between 0.4 and -0.4, the influence of this variable was considered as low; when R or Cramer’s V coefficient was between 0.4 and 0.7 or -0.4 and -0.7 the influence was considered as moderate, when they were greater than 0.7 or lower than -0.7 the influence was considered as strong. Cohen’s D coefficient > 1.5 was considered strong.

Student t-test was performed to compare the means of zinc level in T21 and CTRL populations. We decided to compare the whole populations according to sex and according to age range divided in a pre-puberal group (from 4 to 9 years old), a puberal group (from 9 to 14 years old) and a young adult group (from 14 to 18 years old).

Bivariate correlation was performed between continuous variables and Student’s t-test or Wilcoxon Mann Whitney test were executed between nominal variables to assess an influence of age, sex, fasting state and zinc transporter on zinc values and immunity parameters.

Where this influence was not found, bivariate correlation was used to compare zinc and immunity values, dividing subjects by fasting state. Where an influence was detected, partial correlation was performed.

Chi-squared test was used to search for an association between High/Low levels compared to the median value of zinc and of immunity cells/factors.

### Gene selection

For gene expression analysis, we first performed a bibliographic search on the NCBI PubChem database (https://pubchem.ncbi.nlm.nih.gov) to find genes involved in zinc metabolism. We searched for “Zinc”, “Zinc fingers” and “Zinc fingers transcription factor” in the PubChem toolbar adding as filter “Homo sapiens”. We selected the Gene section and examined all the gene sheets, establishing if they had an effective interaction with zinc molecules.

### Transcriptome mapper for gene expression analysis

Transcriptome Mapper (TRAM) is a validated software that is able to import gene expression data from NCBI Gene Expression Omnibus (GEO) and ArrayExpress databases in tab-delimited text format (34, 35). It allows the integration of all data through the decoding of probes identifiers to gene symbols, through UniGene data parsing (36); TRAM performs an intra- and inter-sample normalization (scaled quantile normalization) of gene expression values and creates a graphical representation of the gene expression profile in two different modes, “Map” or “Cluster”, and assesses the statistical significance of the results by statistical tests. Moreover, TRAM allows the comparison of two biological conditions, in this case “Pool A” or T21 samples and “Pool B” or control samples, identifying critical genomic regions and genes with significant differential expression.

In this work, we used TRAM to analyze differential transcriptome maps of lymphoblastoid cells, thymus and white blood cells already validated (35) focusing on differentially expressed genes (DEGs) implicated in zinc metabolism which are expressed with a T21/CTRL ratio >1.3 or <0.76. We excluded genes whose expression was lower than 1 in both T21 and CTRL groups.

## Results

### Comparison of zinc levels in T21 subjects and control group

First, the Kolmogorov-Smirnov test was performed to check for normal distribution. Excluding one extremely high zinc value (greater than mean + 2 SD) from the total of 217 subjects enrolled, data on blood zinc levels are available from 216 subjects with T21. Results indicated that the data distribution is normal or Gaussian. Indeed, the mean is 13.03 and median is 12.70 (**Table 1**).

**Table 1:** Zinc in children with trisomy 21 (µmol/L).

		tot	M	F	Y<4	4≤y<9	9≤y<14	14≤y<18	y≥18
**Zinc** *µmol/l*	**n**	216	133	83	30	77	59	25	25
	**mean**	13.03	13.37	12.48	12.44	13.49	12.94	12.29	13.27
	**SD**	3.46	3.34	3.61	4.20	3.61	2.99	3.00	3.53

n, numerosity; SD, standard deviation; TOT, total number of subjects; M, male subjects; F, female subjects; Y<4, subjects younger than 4 years old; 4≤Y<9, subjects aged between 4 and 9 years old; 9≤Y<14, subjects aged between 9 and 14 years old; 14≤Y<18, subjects aged between 14 and 18 years old; Y≥18, subjects older than 18 years old.

**Figure 1:**
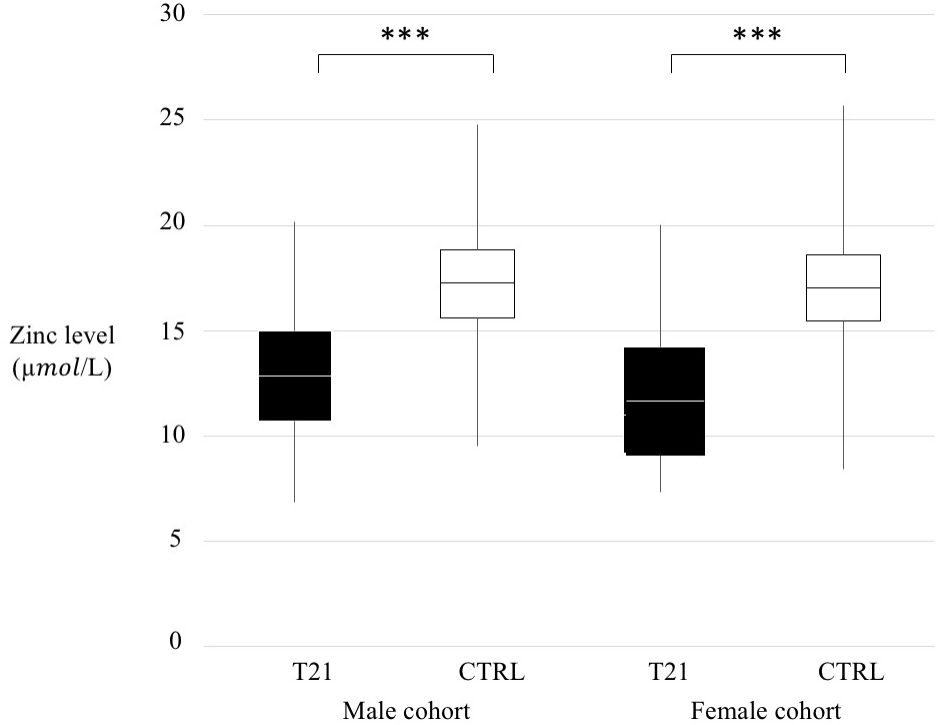
Zinc level comparison trisomy 21 (T21) and control (CTRL) groups according to sex. Black box=T21 subjects, White box= CTRL subjects, ***p < 0.001.

**Figure 2:**
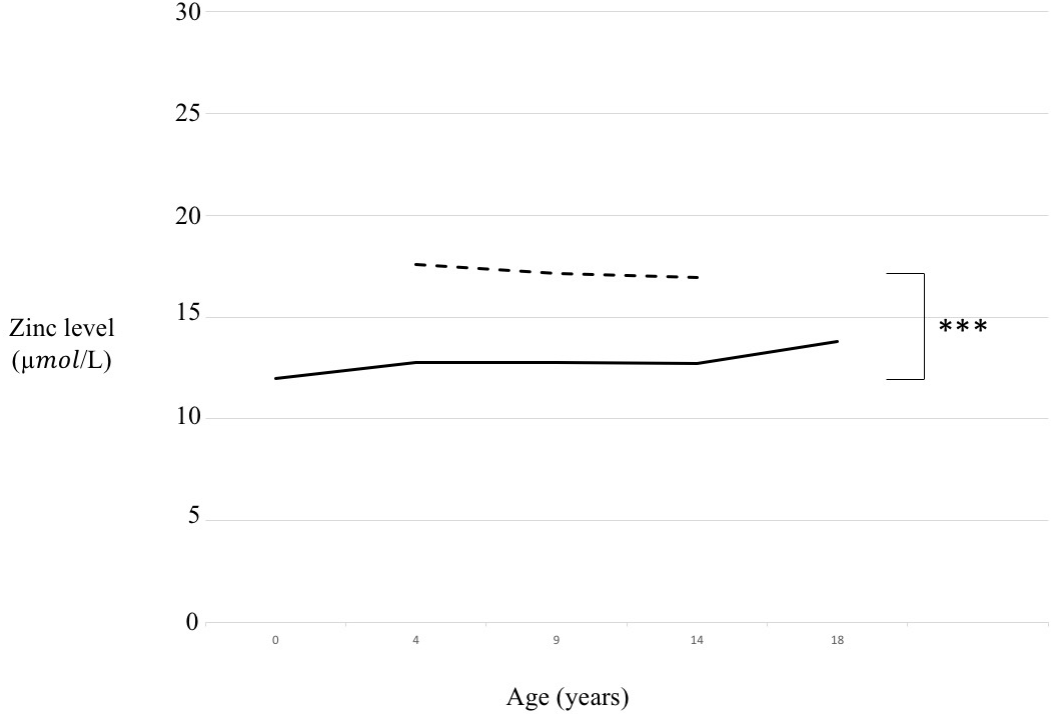
Zinc level comparison in trisomy 21 (T21) and control (CTRL) groups according to age range. Continuous line= T21 subjects, Dotted line= CTRL subjects, ***p < 0.001.

We compared T21 and CTRL whole populations (**Supplementary Table 2**, p < 0.001) according to sex (**Figure 1**) and according to age range divided in a pre-puberal group (from 4 to 9 years old, p < 0.001), a puberal group (from 9 to 14 years old, p < 0.001) and a young adult group (from 14 to 18 years old, p < 0.001) (**Figure 2**).

### Gene expression analysis using TRAM software

From the bibliographic search, we found in total 55 genes involved in zinc metabolism (**Supplementary Table 3**) and we studied their expressions in lymphoblastoid cell lines, in thymus and in white blood cells.

Concerning the lymphoblastoid cell lines, we found that 54 out of 55 selected genes had an available gene expression value and 5 of them were DEGs: 4 genes are overexpressed with a ratio of 3:2, 3 of which belong to metallothionein family and the fourth is *SOD1*; only one (*IK7F3*) is underexpressed with a ratio of 2:3 (**Table 2**; **Supplementary Table 4A**).

**Table 2:** List of differentially expressed genes (DEGs) in a pool of lymphoblastoid cells with trisomy 21 versus a pool of euploid lymphoblastoid cells (Pelleri et al., 2018).

Gene name	Description	Location	Expression in T21	Expression in CTRL	T21/CTRL ratio
*MT1G*	Metallothionein 1G	16q13	63.96	44.24	1.45 ↑
*MT1H*	Metallothionein 1H	16q13	83.80	56.96	1.47↑
*IKZF3*	IKAROS family zinc finger 3	17q12-q21.1	87.95	117.52	0.75↓
*SOD1*	Superoxide dismutase 1	21q22.11	1761.69	1258.93	1.40↑
*MT1HL1*	Metallothionein 1H like 1	1q43	114.50	84.87	1.35↑

T21, Trisomy 21; CTRL, control. ↑= T21/CTRL ratio > 1.3 ↓= T21/CTRL ratio < 0.76.

In thymus, we found that 45 out of 55 selected genes had an available gene expression value and 35 of them were DEGs: 3 genes of the Ikaros family zinc finger (IKZ family) are underexpressed, 2 of which with a ratio of 2:3; 2 genes of the KLF transcription factor family are overexpressed and 1 gene is underexpressed with a 2:3 ratio; 8 genes of the metallothionein family are overexpressed, only one with a ratio of 3:2; 2 metal transcription factor genes are dysregulated with a specular ratio of 3:2 and 2:3; *PRDM10* has a 2:3 ratio; 3 SLC30 family genes out of 5 are overexpressed, 2 of which with a 3:2 ratio, and 2 gene out of 5 is underexpressed with a 1:2 ratio; 3 genes of the SLC39 genes are overexpressed, 1 of which with a 3:2 ratio and 5 are underexpressed, 2 of which with a 2:3 ratio; *BCL6*, *SOD1* and *ZHX3* are overexpressed with a ratio of 3:2; *TF* is overexpressed (**Table 3**; **Supplementary Table 4B**).

**Table 3:** List of Differentially Expressed Genes (DEGs) in a pool of thymus cells with trisomy 21 versus a pool of euploid thymus cells (Pelleri et al., 2018).

Gene name	Description	Location	Expression in T21	Expression in CTRL	T21/CTRL ratio
*BCL6*	BCL6 transcription repressor	3q27.3	400.17	229.97	1.74↑
*IKZF1*	IKAROS family zinc finger 1	7p12.2	37.43	58.35	0.64↓
*IKZF2*	IKAROS family zinc finger 2	2q34	82.49	203.7	0.40↓
*IKZF3*	IKAROS family zinc finger 3	17q12-q21.1	8.36	11.97	0.70↓
*KLF10*	KLF transcription factor 10	8q22.3	34.56	6.56	5.27↑
*KLF13*	KLF transcription factor 13	15q13.3	360.04	620.15	0.58↓
*KLF2*	KLF transcription factor 2	19p13.11	306.36	172.02	1.78↑
*MT1A*	Metallothionein 1A	16q13	416.48	193.39	2.15↑
*MT1B*	Metallothionein 1B	16q13	258.996	75.97	3.41↑
*MT1E*	Metallothionein 1E	16q13	197.50	74.02	2.67↑
*MT1F*	Metallothionein 1F	16q13	18.31	9.81	1.87↑
*MT1G*	Metallothionein 1G	16q13	166.99	71.47	2.34↑
*MT1H*	Metallothionein 1H	16q13	281.66	132.01	2.13↑
*MT1M*	Metallothionein 1M	16q13	47.72	13.23	3.61↑
*MT1X*	Metallothionein 1X	16q13	228.96	100.91	2.27↑
*MT2A*	Metallothionein 2A	16q13	978.21	330.40	2.96↑
*MTF1*	Metal regulatory transcription Factor 1	1p34.3	78.52	53.59	1.47↑
*MTF2*	Metal response element binding transcription factor 2	1p22.1	43.04	69.80	0.62↓
*PRDM10*	PR/SET domain 10	11q24.3	19.59	33.36	0.59↓
*SLC30A1*	Solute carrier family 30 member 1	1q32.3	13.38	7.69	1.74↑
*SLC30A2*	Solute carrier family 30 member 2	1p36.11	4.47	1.91	2.34↑
*SCL30A4*	Solute carrier family 30 member 4	15q21.1	5.37	3.84	1.40↑
*SCL30A5*	Solute carrier family 30 member 5	5q13.1-q13.2	16.86	30.55	0.55↓
*SLC30A7*	Solute carrier family 30 member 7	1p21.2	9.69	19.53	0.50↓
*SLC39A10*	Solute carrier family 39 member 10	2q32.3	12.61	22.97	0.55↓
*SLC39A11*	Solute carrier family 39 member 11	17q24.3-q25.1	11.74	18.83	0.62↓
*SLC39A14*	solute carrier family 39 member 14	8p21.3	64.67	22.37	2.89↑
*SLC39A2*	Solute carrier family 39 member 2	14q11.2	1.82	3.94	0.46↓
*SLC39A5*	Solute carrier family 39 member 5	12q13.3	2.66	1.97	1.35↑
*SLC39A7*	Solute carrier family 39 member 7	6p21.32	19.66	32.48	0.61↓
*SLC39A8*	Solute carrier family 39 member 8	4q24	39.64	77.21	0.51↓
*SLC39A9*	Solute carrier family 39 member 9	14q24.1	5.57	3.16	1.76↑
*SOD1*	Superoxide dismutase 1	21q22.11	750.75	488.35	1.54↑
*TF*	transferrin	3q22.1	38.64	11.10	3.48↑
*ZHX3*	Zinc finger and homeboxes 3	20q12	65.84	40.28	1.63↑

T21, Trisomy 21; CTRL, control. ↑= T21/CTRL ratio > 1.3 ↓= T21/CTRL ratio < 0.76.

In white blood cells, finally, we found that 41 out of 55 selected genes had an available gene expression value. Only one DEG is underexpressed (*IKZF2* gene, 2:3 ratio) and 2 DEGs overexpressed with a ratio of 3:2 (**Table 4**; **Supplementary Table 4C**).

**Table 4:** List of Differentially Expressed Genes (DEGs) in a pool of white blood cells with trisomy 21 versus a pool of euploid white blood cells (Pelleri et al., 2018).

Gene name	Description	Location	Expression in T21	Expression in CTRL	T21/CTRL ratio
*IKZF2*	IKAROS family zinc finger 2	2q34	39.25	56.57	0.69↓
*MT1E*	Metallothionein 1E	16q13	160.14	102.14	1.57↑
*MT2A*	Metallothionein 2A	16q13	816.53	604.32	1.35↑

T21, Trisomy 21; CTRL, control. ↑= T21/CTRL ratio > 1.3 ↓= T21/CTRL ratio < 0.76.

To recap, both thymus and WBC had an overexpression of *MT1E* gene and *MT2A* gene and an underexpression of *IKZF2* gene while both thymus and of lymphoblastoid cells had an overexpression of *MT1G* gene, *MT1H* gene and *SOD1* and un underexpression of *IKZF3* gene; lymphoblastoid cells and WBC had no DEG in common. None of the selected genes is differentially expressed (> 1.3 or 0.76 in all the three selected transcriptome maps. *SOD1* is actually expressed in all the three maps but in the T21/CTRL white blood cell map its expression ratios is 1.29 (thus not in our defined differentially expressed range).

### Correlation between zinc level and immunity values in subjects with T21

Zinc values and immunity parameter values were compared in the population with T21. First of all, the influence of age, sex, fasting state and value of zinc transporters (albumin, α2-macroglobulin and transferrin) at the time of blood draw was investigated in order to know if, considering our data, any correlation between zinc levels and immunity state is a simple bivariate or a partial correlation. As shown in **Supplementary Tables 5**, **6**, zinc level, white blood cells (WBC) level and lymphocytes B level are associated with fasting state. We excluded 7 subjects without information on fasting state for the subsequent analyses concerning these metabolites. Lymphocytes B level together with immunoglobulins G (IgG) and immunoglobulins A (IgA) showed a moderate correlation with age at blood drawn, IgG and immunoglobulins M (IgM) are associated with sex and IgG showed a moderate correlation with α2-macroglobulin levels (**Supplementary Tables 5**, **6**).

Later, we searched for a correlation between blood zinc values and immunity state, taking as representative of the latter values of WBC, neutrophils, lymphocytes, monocytes, eosinophils, basophils, values from lymphocytes typing and values of IgG, IgA and IgM.

When a linear correlation was studied, no strong correlation emerged. We found a significant correlation with weak strength in fasting subjects between zinc level and lymphocytes T CD4+ cells (r=-0.244, p=0.043), lymphocytes B cells (r=-0.256, p=0.020) and IgA (r=-0.232, p=0.028) and non-fasting between zinc level and natural killer cells (r=0.244, p=0.015) (**Supplementary Table 7A, B**).

Furthermore, Chi-squared test was performed, transforming continuous variables into nominal variable “High” and “Low” in relation to the median value. In this analysis we did not find any statistically significant alterations (**Supplementary Tables 8A, B**).

## Discussion

One of the clinical comorbidities of subjects with DS is the alteration of the immune system and zinc is a chemical element known to have an important role in immune response. The first aim of our work was to measure zinc concentration level in our population with T21. Considering the literature, zinc levels in subjects with T21 is lower compared to the general population; this trend is confirmed from the comparison between our population of 216 children with T21 and a control population taken from the previous literature (33). The difference between the two groups is not influenced by sex and it is not limited to a single age group, but it is present for almost the entire duration of the childhood period. This implies an altered zinc metabolism in subjects with T21, which confirms a metabolic alteration. Moreover, the mean ratio between plasma zinc level in T21 and control groups is 0.75, thus with a 2:3 ratio consistent with a chromosomal dosage effect due to the presence of a third chromosome 21. Remarkably, the level of other several metabolites in children with T21 has been found to be consistent with a 3:2 or 2:3 ratio when compared to the normal level (37, 38), supporting a chromosomal dosage effect and indicating that the third chromosome 21 causes specific alterations in several pathways triggering cascades of events in proportion to the dosage of the genes it contains.

We later searched for any difference between general population and T21 population in gene expression in lymphoblastoid cells, thymus and white blood cells, using transcriptome maps from subjects with trisomy 21 and control group subjects available in the literature. The *SOD1* gene, located on Hsa21, is found overexpressed in the thymus and lymphoblastoid cell transcriptome maps analyzed in this work, with a consistent expression ratio of 3:2, confirming the previous literature (21, 39). We can speculate that, in subjects with DS, an excess of the transcript and thus protein, may lead to the sequestration of zinc and a subsequent decrease in serum of 2:3 ratio.

The analysis of three transcriptome maps reported the overexpression of the genes encoding for the metallothionein family, a group of small proteins that chelate metal ions through metal-thiolate bonds with cysteine residues and that have a role in cellular zinc homeostasis and metal detoxification. Their expression is induced by metals via multiple metal-responsive elements (MREs) present in the metallothionein gene 5´-regulatory regions. As a gene family, they show developmentally regulated expression patterns and cell-type-specific regulation (40). The increased expression of these genes leads to a higher presence of metallothionein proteins that may cause a sequester inside cells of the circulating zinc. Not all gene expression ratios respect the 3:2 ratio, suggesting that the alteration of these genes is due to a chain of regulatory events.

Concerning the Ikaros zinc finger gene family, we found expression of genes belonging to this gene family in lymphoblastoid and thymus cell lines and for all genes a lower expression was found, with a prevalence of 2:3 gene expression ratio. Ikaros proteins are important for lymphocyte development and other physiological processes. Clinical studies found that genetic alterations in Ikaros genes correlates to a poor outcome in high-risk acute lymphoblastic leukemia (ALL) patients and it was highlighted that the 83.7% of patients with Philadelphia chromosome-carrying ALL had partially or total decrease of *IKZF1* gene expression (41). Our results are consistent with a possible alteration of the immune system in subjects with DS (42–44).

Focusing on *SLC* genes, whose role is to exporting zinc out or sequestrating cytoplasmic zinc into intracellular compartments when cellular zinc levels are elevated (SLC30 proteins) or importing the ion when cellular zinc is depleted (SLC39 proteins), 4 genes of the *SLC30* family and 8 genes of the *SLC39* family are DEGs. A higher prevalence of overexpressed *SLC30* genes and underexpressed *SLC39* genes is found. These data, found in the thymus transcriptome map, may suggest that there is an increased intracellular zinc level that could cause a decrease of extracellular zinc concentration.

At least, we searched for possible correlation between zinc levels and immune cell levels also measured in our T21 population. Considering correlation analyses with immunity state parameters, in our data, zinc level seem to not be correlated or associated with immunity data analyzed with few exceptions where strength is considerable as “low”. Our data suggest that zinc level is not associated with the levels of immunity cells or proteins analyzed so the main role of this ion could be played in the alteration of immunity cell function as also previously hypothesized (11).

In addition, zinc levels could also influence other systems that should be analyzed such as thyroid function or cognitive profile (45–47). For these reasons, zinc supplementation should be considered.

The American Academy of Pediatrics suggests a zinc supplementation trial when zinc deficiency is suspected, even though in children the proper daily dose may be difficult to determine, particularly during periods of rapid growth and there is a risk of excessive doses causing copper deficiency. The supplement can be administered as an oral solution of zinc acetate (a dose of 30 mg of zinc acetate in 5 mL of water should usually be adequate). Zinc fortification of food staples may also increase the zinc status of high-risk populations but seems less effective if other micronutrients are added to the food staple in addition to zinc (48).

Concluding, zinc level is confirmed to be lower in subjects with T21 and for the first time, we have demonstrated a 2:3 ratio (compared to the normal level) consistent with a chromosomal dosage effect. In particular, the alteration of some genes’ expression could explain low levels of zinc in blood and high levels of intracellular zinc (23). Future perspectives regarding this topic include increasing the number of trisomic subjects studied and study zinc influence on other systems besides the immune system, such as thyroid or nervous.

## Data availability statement

The datasets presented in this study can be found in online repositories. The names of the repository/repositories and accession number(s) can be found in the article/**Supplementary Material**.

## Ethics statement

The studies involving humans were approved by Comitato Etico Area Vasta Emilia Centro. The studies were conducted in accordance with the local legislation and institutional requirements. Written informed consent for participation in this study was provided by the participants’ legal guardians/next of kin.

## Author contributions

GR: Data curation, Investigation, Supervision, Writing – original draft. CL: Data curation, Writing – review & editing. MS: Formal analysis, Methodology, Software, Writing – review & editing. MCP: Formal analysis, Methodology, Writing – review & editing. MCa: Formal analysis, Validation, Writing – review & editing. AP: Formal analysis, Software, Writing – review & editing. MCi: Formal analysis, Writing – review & editing. VM: Formal analysis, Writing – review & editing. LV: Formal analysis, Investigation, Writing – review & editing. GS: Data curation, Writing – review & editing. LTC: Data curation, Writing – review & editing. GLP: Data curation, Writing – review & editing. PS: Conceptualization, Project administration, Writing – review & editing. FC: Formal analysis, Writing – review & editing. BV: Conceptualization, Writing – review & editing. FA: Conceptualization, Writing – review & editing.
