# Supplementary material for: Zinc metabolism and its role in immunity status in subjects with trisomy 21: chromosomal dosage effect
Source: Front Immunol. 2024 Apr 17;15:1362501. doi: 10.3389/fimmu.2024.1362501 (PMC11061464; doi:10.3389/fimmu.2024.1362501)
Supplement: Supplementary file 2 [file DataSheet_2.docx]

**Instructions to obtain standard deviation from 95% confidence interval**

A normal distribution and number of cases was described for the control population, mean and 95% confidence interval for male and female of each age were reported (López 1997). Since standard deviation was not available, it was calculated from the confidence interval for each subgroup~~,~~ using the formula:

$$SD = \frac{\left( 95\% CI-\bar{x} \right)}{2}$$

where SD=standard deviation, 95% CI=one of the two 95% confidence interval’s ends, $\bar{x}$=mean.

Larger subgroups were created grouping together all males of all ages, all females of all ages, both males and females of each age group and now grouping the population into ranges of 4-9, 9-14 and 14-18 year old subjects and, in the end, all males and all females of all ages. For each of these larger subgroups, the number of cases was calculated by adding the number of cases of the subgroups from which it was composed; mean was calculated by performing a weighted mean, using number of cases as weight; after these two data were obtained, standard deviation for each larger subgroup was calculated by the following formula:

$${SD}^{2}= \frac{\sum_{i=1}^{k} [{SD}_{i}^{2}(n_{i}-1)]+ \sum_{i=1}^{k} [n_{i}\left( \bar{x}_{i}- \bar{x} \right)^{2}]}{\left( \sum_{i=1}^{k} n_{i} \right)-1}$$

where SD=standard deviation, k=number of subgroups, ${SD}_{i}$=standard deviation of the i-th subgroup, $n_{i}$=numerosity of the i-th subgroup, $\bar{x}_{i}$=weighted mean of the i-th subgroup, $\bar{x}$=weighted mean of the whole sample.
