# Supplementary material for: Zinc metabolism and its role in immunity status in subjects with trisomy 21: chromosomal dosage effect
Source: Front Immunol. 2024 Apr 17;15:1362501. doi: 10.3389/fimmu.2024.1362501 (PMC11061464; doi:10.3389/fimmu.2024.1362501)
Supplement: Supplementary file 3 [file Table_1.docx]

|  | | tot | M | F | 4≤y<9 | 9≤y<14 | 14≤y<18 |
| --- | --- | --- | --- | --- | --- | --- | --- |
| Zinc  *(µmol/L)* | n | 3668 | 1891 | 1777 | 1147 | 1383 | 1138 |
|  | mean | 17.20 | 17.34 | 17.06 | 17.56 | 17.13 | 16.94 |
|  | SD | 2.42 | 2.47 | 2.35 | 2.44 | 2.35 | 2.43 |

**Supplementary Table 1.** *Zinc in children without trisomy 21 (µmol/L).*

López 1997 reported number of cases, average values and 95% confidence intervals (CI) for male and female groups of each age. Standard deviation (SD) was calculated starting from CI. Number (n), mean and SD for the total (TOT) population, the total male (M) population, the total female (F) population and each interval of ages was calculated starting from the available data. 4≤Y<9=subjects aged between 4 and 9 years old, 9≤Y<14=subjects aged between 9 and 14 years old, 14≤Y<18=subjects aged between 14 and 18 years old
