# Supplementary material for: Zinc metabolism and its role in immunity status in subjects with trisomy 21: chromosomal dosage effect
Source: Front Immunol. 2024 Apr 17;15:1362501. doi: 10.3389/fimmu.2024.1362501 (PMC11061464; doi:10.3389/fimmu.2024.1362501)
Supplement: Supplementary file 4 [file Table_2.docx]

|  | **TOT** | | | **M** | | | **F** | | | **4≤y<9** | | | **9≤y<14** | | | **14≤y<18** | | |
| --- | --- | --- | --- | --- | --- | --- | --- | --- | --- | --- | --- | --- | --- | --- | --- | --- | --- | --- |
|  | **T21** | **CTRL** | **p** | **T21** | **CTRL** | **p** | **T21** | **CTRL** | **p** | **T21** | **CTRL** | **p** | **T21** | **CTRL** | **p** | **T21** | **CTRL** | **p** |
| **mean** | 13.03 | 17.20 | **<0.001** | 13.37 | 17.34 | **<0.001** | 12.48 | 17.06 | **<0.001** | 13.49 | 17.56 | **<0.001** | 12.94 | 17.13 | **<0.001** | 12.29 | 16.94 | **<0.001** |
| **SD** | 3.46 | 2.42 |  | 3.34 | 2.47 |  | 3.61 | 2.35 |  | 3.61 | 2.44 |  | 2.99 | 2.35 |  | 3.00 | 2.43 |  |

**Supplementary Table 2.** *Comparison between zinc values (µmol/L) of children with trisomy 21 and controls.*

TOT=total subjects, M=male subjects, F=female subjects, 4≤Y<9=subjects aged between 4 and 9 years old, 9≤Y<14=subjects aged between 9 and 14 years old, 14≤Y<18=subjects aged between 14 and 18 years old, T21=Trisomy 21, CTRL=controls, p=p-value, SD=standard deviation. Statistically significant values are highlighted in bold (p-value<0.05).
