# Supplementary material for: Zinc metabolism and its role in immunity status in subjects with trisomy 21: chromosomal dosage effect
Source: Front Immunol. 2024 Apr 17;15:1362501. doi: 10.3389/fimmu.2024.1362501 (PMC11061464; doi:10.3389/fimmu.2024.1362501)
Supplement: Supplementary file 7 [file Table_5.docx]

|  | | **Age** | **Sex** | **Fasting state** | **Albumin** | **α2-macroglobulin** | **Transferrin** |
| --- | --- | --- | --- | --- | --- | --- | --- |
| **Zinc**  *(µmol/L).* | n | 216 | 216 | **209** | 208 | 212 | 174 |
|  | p | 0.528 | 0.068 | **0.007** | **<0.001** | 0.526 | 0.110 |
|  | R/D | -0.043 | 3.44 | 3.35 | 0.232 | -0.044 | 0.147 |

**Supplementary Table 5.** *Comparison between zinc level and age, gender, fasting state and zinc transporters levels*.

n=number of subjects, p=significance, R=Pearson’s coefficient, D=Cohen’s coefficient. For continuous variables (age, albumin, α2-macroglobulin, transferrin) bivariate correlation has been performed and p-value and R are reported. For categorical variables (gender and fasting state) t-test has been performed and p-value and Cohen’s D are reported. Statistically significant values are highlighted in bold (p-value<0.05).
