# Supplementary material for: Zinc metabolism and its role in immunity status in subjects with trisomy 21: chromosomal dosage effect
Source: Front Immunol. 2024 Apr 17;15:1362501. doi: 10.3389/fimmu.2024.1362501 (PMC11061464; doi:10.3389/fimmu.2024.1362501)
Supplement: Supplementary file 8 [file Table_6.docx]

|  | | **Age** | **Sex** | **Fasting state** | **Albumin** | **α2-macroglobulin** | **Transferrin** |
| --- | --- | --- | --- | --- | --- | --- | --- |
| **White blood cells**  (10^3^/mmc) | n | 212 | 212 | 205 | 204 | 208 | 171 |
|  | p | **0.003** | 0.858 | **0.008** | 0.064 | **<0.001** | 0.963 |
|  | R/D | -0.202 | 2.15 | 2.06 | 0.365 | 0.297 | 0.004 |
| **Neutrophils***  (10^3^/mmc) | n | 210 | 210 | 204 | 202 | 206 | 169 |
|  | p | 0.578 | 0.631 | 0.097 | 0.160 | **0.004** | 0.404 |
|  | R/D | -0.039 | 0.48 | 1.52 | 0.099 | 0.199 | -0.065 |
| **Lymphocytes**  (10^3^/mmc) | n | 212 | 212 | 205 | 204 | 208 | 171 |
|  | p | **<0.001** | 0.911 | **0.036** | 0.606 | **0.014** | **0.001** |
|  | R/D | -0.303 | 0.87 | 0.85 | 0.036 | 0.170 | 0.245 |
| **Monocytes***  (10^3^/mmc) | n | 210 | 210 | 203 | 202 | 206 | 169 |
|  | p | **0.036** | 0.322 | 0.145 | 0.928 | **<0.001** | 0.766 |
|  | R/D | -0.145 | 0.990 | 0.14 | -0.006 | 0.280 | 0.023 |
| **Eosinophils***  (10^3^/mmc) | n | 211 | 211 | 204 | 203 | 207 | 170 |
|  | p | **<0.001** | 0.696 | 0.201 | 0.252 | 0.220 | 0.524 |
|  | R/D | -0.233 | 0.388 | 0.15 | -0.081 | 0.086 | -0.049 |
| **Basophils***  (10^3^/mmc) | n | 212 | 212 | 205 | 204 | 208 | 171 |
|  | p | 0.187 | 0.603 | 0.353 | 0.449 | 0.271 | 0.280 |
|  | R/D | -0.091 | 0.517 | 0.04 | 0.053 | 0.077 | 0.083 |
| **T cells**  (10^3/^mmc) | n | 183 | 183 | 177 | 176 | 181 | 144 |
|  | p | **0.011** | 0.553 | 0.096 | 0.763 | 0.209 | 0.061 |
|  | R/D | -0.188 | 664.66 | 684.54 | 0.023 | 0.094 | 0.156 |
| **CD4+**  **T helper cells**  (10^3/^mmc) | n | 182 | 182 | 175 | 174 | 179 | 143 |
|  | p | **0.001** | 0.070 | 0.054 | 0.248 | 0.153 | 0.210 |
|  | R/D | -0.240 | 315.18 | 330.02 | 0.063 | 0.107 | 0.106 |
| **CD8+**  **cytotoxic T cells***  (10^3/^mmc) | n | 182 | 182 | 176 | 175 | 180 | 143 |
|  | p | 0.765 | 0.992 | 0.261 | 0.153 | 0.924 | **0.048** |
|  | R/D | -0.022 | -0.005 | 413.10 | 0.108 | -0.007 | 0.166 |
| **Natural killer cells***  (10^3/^mmc) | n | 180 | 180 | 175 | 173 | 178 | 141 |
|  | p | 0.164 | 0.928 | 0.791 | **0.002** | 0.956 | 0.427 |
|  | R/D | -0.104 | -0.092 | 175.44 | 0.236 | -0.004 | 0.067 |
| **B Cells**  (10^3/^mmc) | n | 183 | 183 | 177 | 176 | 181 | 144 |
|  | p | **<0.001** | 0.413 | **0.006** | **0.008** | **<0.001** | **0.023** |
|  | R/D | -0.643 | 139.05 | 134.96 | -0.200 | 0.302 | 0.189 |
| **IgG**  (mg/dl) | n | 207 | 207 | 201 | 199 | 203 | 165 |
|  | p | **<0.001** | **0.006** | 0.050 | 0.600 | **<0.001** | 0.139 |
|  | R/D | 0.452 | 297.37 | 282.48 | 0.037 | -0.498 | -0.116 |
| **IgA***  (mg/dl) | n | 210 | 210 | 204 | 202 | 206 | 168 |
|  | p | **<0.001** | 0.317 | 0.124 | 0.136 | **<0.001** | **0.002** |
|  | R/D | 0.617 | -0.999 | 91.50 | 0.105 | -0.367 | -0.239 |
| **IgM***  (mg/dl) | n | 207 | 207 | 201 | 199 | 203 | 165 |
|  | p | 0.713 | **<0.001** | 0.177 | **0.018** | 0.361 | 0.457 |
|  | R/D | 0.026 | -4.755 | 28.01 | -0.167 | -0.064 | -0.058 |

**Supplementary Table 6.** *Comparison between immunity values and age, gender, fasting state and zinc transporter levels*.

n=number of subjects, p=significance, R=Pearson’s coefficient, D=Cohen’s coefficient. For continuous variables (age, albumin, α2-macroglobulin, transferrin) bivariate correlation has been performed and p-value and R are reported. For categorical variables (gender and fasting state) t-test has been performed and p-value and Cohen’s D are reported. Statistically significant values are highlighted in bold (p-value<0.05). *=Mann Whitney U test used to analyze sex and fasting state influence
