# Supplementary material for: Zinc metabolism and its role in immunity status in subjects with trisomy 21: chromosomal dosage effect
Source: Front Immunol. 2024 Apr 17;15:1362501. doi: 10.3389/fimmu.2024.1362501 (PMC11061464; doi:10.3389/fimmu.2024.1362501)
Supplement: Supplementary file 9 [file Table_7.docx]

|  | | | **White blood cells**  (10^3^/mmc)  (n=205) | **Neutrophils**  (10^3^/mmc)  (n=204) | **Lymphocytes**  (10^3^/mmc)  (n=205) | **Monocytes**  (10^3^/mmc)  (n=203) | **Eosinophils**  (10^3^/mmc)  (n=204) | **Basophils**  (10^3^/mmc)  (n=205) |
| --- | --- | --- | --- | --- | --- | --- | --- | --- |
| **Zinc**  *(µmol/L)* | **Fasting** | n | 91 | 91 | 91 | 90 | 91 | 91 |
|  |  | p | 0.345 | 0.415 | 0.619 | 0.535 | 0.495 | 0.806 |
|  |  | R | -0.100 | -0.086 | -0.053 | -0.066 | -0.066 | -0.026 |
|  | **Not fasting** | n | 114 | 113 | 114 | 113 | 113 | 114 |
|  |  | p | 0.246 | 0.059 | 0.498 | 0.434 | 0.849 | 0.307 |
|  |  | R | -0.109 | -0.178 | 0.064 | 0.074 | 0.018 | 0.097 |

**Supplementary Table 7a.** *Comparison between zinc level and immunity values in fasting and not fasting subjects*.

n=number, p=significance, R=Pearson correlation number. Statistically significant values are highlighted in bold (p-value < 0.05).

|  | | | **T cells**  (10^3^/mmc)  (n=177) | **CD4+ T helper cells**  (10^3^/mmc)  (n=175) | **CD8+ cytotoxic T cells**  (10^3^/mmc)  (n=176) | **Natural killer**  (10^3^/mmc)  (n=175) | **B cells***  (10^3^/mmc)  (n=177) | **Immunoglobulin G**^$^  (mg/dL)  (n=201) | | **Immunoglobulin A***  (mg/dL)  (n=204) | **Immunoglobulin M**  (mg/dL)  (n=201) | |
| --- | --- | --- | --- | --- | --- | --- | --- | --- | --- | --- | --- | --- |
|  |  |  |  |  |  |  |  | **M**  (n=124) | **F**  (n=77) |  | **M**  (n=124) | **F**  (n=77) |
| **Zinc**  *(µmol/L)* | **Fasting** | n | 78 | 77 | 78 | 77 | 78 | 52 | 38 | 91 | 52 | 38 |
|  |  | p | 0.395 | 0.053 | 0.615 | 0.501 | **0.020** | 0.875 | 0.620 | **0.028** | 0.333 | 0.729 |
|  |  | R | -0.098 | -0.222 | -0.058 | 0.078 | -0.256 | -0.023 | 0.086 | -0.232 | -0.137 | -0.058 |
|  | **Not fasting** | n | 99 | 98 | 98 | 98 | 99 | 72 | 39 | 113 | 72 | 39 |
|  |  | p | 0.233 | 0.709 | 0.215 | **0.015** | 0.939 | 0.751 | 0.502 | 0.642 | 0.475 | 0.191 |
|  |  | R | 0.121 | 0.038 | 0.126 | 0.244 | 0.008 | 0.039 | -0.116 | -0.044 | -0.085 | -0.214 |

**Supplementary Table 7b.** *Comparison between zinc level and immunity values in fasting and not fasting subjects*.

n=number, p=significance, R=Pearson correlation number. * partial correlation corrected by age. $ partial correlation corrected by age and α2-macroglobulin. Statistically significant values are highlighted in bold (p-value<0.05).
