# Supplementary material for: Zinc metabolism and its role in immunity status in subjects with trisomy 21: chromosomal dosage effect
Source: Front Immunol. 2024 Apr 17;15:1362501. doi: 10.3389/fimmu.2024.1362501 (PMC11061464; doi:10.3389/fimmu.2024.1362501)
Supplement: Supplementary file 10 [file Table_8.docx]

|  | | | **White blood cells H/L**  (n=205) | **Neutrophils H/L**  (n=204) | **Lymphocytes H/L**  (n=205) | **Monocytes H/L**  (n=203) | **Eosinophils H/L**  (n=204) | **Basophils H/L**  (n=205) |
| --- | --- | --- | --- | --- | --- | --- | --- | --- |
| **Zinc**  ***H/L*** | **Fasting** | n | 91 | 91 | 91 | 90 | 91 | 91 |
|  |  | p | 0.626 | 0.891 | 0.774 | 0.654 | 0.242 | 0.105 |
|  |  | V | 0.101 | 0.014 | 0.030 | 0.047 | 0.123 | 0.170 |
|  | **Not fasting** | n | 114 | 113 | 114 | 113 | 113 | 114 |
|  |  | p | 0.255 | **0.040** | 0.614 | 0.617 | 0.397 | 0.737 |
|  |  | V | 0.107 | 0.194 | 0.047 | 0.047 | 0.080 | 0.031 |

**Supplementary Table 8a.** *Comparison between zinc level and immunity values in fasting and not fasting subjects*.

H/L=High/Low value compared to median value, N=number of subjects, p=significance, V=Cramer’s V coefficient. Statistically significant values are highlighted in bold (p-value<0.05).

|  | | | **T cells**  **H/L**  (n=177) | **CD4+ T helper cells H/L**  (n=175) | **CD8+ cytotoxic T cells H/L**  (n=176) | **Natural killer H/L**  (n=175) | **B cells***  **H/L**  (n=177) | **Immunoglobulin G**^$^  **H/L**  (n=201) | | **Immunoglobulin A***  **H/L**  (n=204) | **Immunoglobulin M**  **H/L**  (n=201) | |
| --- | --- | --- | --- | --- | --- | --- | --- | --- | --- | --- | --- | --- |
|  |  |  |  |  |  |  |  | **M**  (n=124) | **F**  (n=77) |  | **M**  (n=124) | **F**  (n=77) |
| **Zinc H/L** | **Fasting** | n | 78 | 77 | 78 | 77 | 78 | 52 | 38 | 91 | 52 | 38 |
|  |  | p | 0.427 | 0.127 | 1.000 | 0.964 | 0.698 | 0.830 | 0.205 | 0.198 | 0.160 | 0.435 |
|  |  | V | 0.090 | 0.174 | < 0.001 | 0.005 | 0.044 | 0.030 | 0.206 | 0.135 | 0.195 | 0.127 |
|  | **Not fasting** | n | 99 | 98 | 98 | 98 | 99 | 72 | 39 | 113 | 72 | 39 |
|  |  | p | 0.872 | 0.501 | 0.108 | 0.749 | 0.400 | 0.526 | 0.648 | 0.810 | 0.109 | **0.043** |
|  |  | V | 0.016 | 0.068 | 0.163 | 0.032 | 0.085 | 0.075 | 0.073 | 0.023 | 0.189 | 0.324 |

**Supplementary Table 8b.** *Comparison between zinc level and immunity values in fasting and not fasting subjects*.

H/L=High/Low value compared to median value, N=number of subjects, p=significance, V=Cramer’s V coefficient. Statistically significant values are highlighted in bold (p-value<0.05).
